# Genome Sequence of the Boron-Tolerant and -Requiring Bacterium *Bacillus boroniphilus*

**DOI:** 10.1128/genomeA.00935-13

**Published:** 2014-01-02

**Authors:** Bekir Çöl, Zeynep Özkeserli, Dibyendu Kumar, Hilal Özdağ, Yeşim D. Alakoç

**Affiliations:** Muğla SK University, Science Faculty, Department of Biology, Kötekli, Muğla, Turkeya; Ankara University, Biotechnology Institute, Central Laboratory, Tandoğan, Ankara, Turkeyb; Waksman Institute of Microbiology, Piscataway, New Jersey, USAc

## Abstract

*Bacillus boroniphilus* is a highly boron-tolerant bacterium that also requires this element for its growth. The complete genome sequence of *B. boroniphilus* was determined by a combination of shotgun sequencing and paired-end sequencing using 454 pyrosequencing technology. A total of 84,872,624 reads from shotgun sequencing and a total of 194,092,510 reads from paired-end sequencing were assembled using Newbler 2.3. The estimated size of the draft genome is 5.2 Mb.

## GENOME ANNOUNCEMENT

Boron is an essential micronutrient for plants, required primarily for maintaining the integrity of cell walls (1). As an ultratrace element, it is also necessary for the optimal health of some animals (2). It was initially reported that B is essential for some cyanobacteria (3). Moreover, it is known to be toxic for living cells when present above a certain threshold (4). However, in 2007, a B-tolerant and -requiring bacterium was isolated from a naturally high-B-containing area in Kütahya, Turkey. The so-called *Bacillus boroniphilus* requires B for its growth and also can tolerate up to 450 mM boric acid (5). This feature makes it an excellent candidate for being a model organism to investigate the boron-bacterium relationship using molecular and biochemical approaches.

We received the *B. boroniphilus* DSM 17376 from the German Collection of Microorganisms and Cell Culture (DZMZ). The bacterium was grown with Difco sporulation medium (DSM) 92 with 50 mM boric acid (30°C at 200 rpm). The genomic DNA was isolated with the Promega Wizard Genomic DNA purification kit. The complete genome sequence of *B. boroniphilus* was determined by a combination of a shotgun sequencing and a paired-end sequencing run using 454 pyrosequencing technology on a GS FLX Titanium platform.

A total of 84,872,624 reads from shotgun sequencing and a total of 194,092,510 reads from paired-end sequencing were assembled using Newbler 2.3, generating three large and 10 small scaffolds. The annotation was performed using both the NCBI-PGAAP and RAST annotation servers (6, 7).

The draft genome of *B. boroniphilus* contains 4,650,916 bases in 13 scaffolds, excluding gaps. The genome comprises 4,538 predicted coding sequences, 61 tRNAs, and 4 rRNAs genes. The G+C content of the genome was found to be 41.35%. The predicted coding sequences were found to be in three COG categories (information storage and processing, metabolism, and cellular processes and signaling). RAST annotation results revealed 94 activities that belong to the group of stress response genes, which might be involved in providing the extremophile strain of *B. boroniphilus* to tolerate or even perhaps require B. Among these genes, those for osmotic stress (4), oxidative stress (40), heat shock (15), detoxification (9), and stress response genes with no category (33) are particularly striking. Furthermore, it was found that a relatively significant number of the annotated genes were classified into the “poorly characterized” COG groups, which indicates that there is still much to study on the genome of this organism. Therefore, the genome of *B. boroniphilus* will likely shed light on the studies regarding boron biology, stress phenotypes, and beyond.

### Nucleotide sequence accession number.

This genome sequence has been deposited at GenBank with the accession no. AWXY00000000.
